# Experimental steering of electron microscopy studies using prior X-ray computed tomography

**DOI:** 10.1016/j.ultramic.2019.03.002

**Published:** 2019-06

**Authors:** Tobias Starborg, James D.B. O'Sullivan, Claudia Martins Carneiro, Julia Behnsen, Kathryn J. Else, Richard K. Grencis, Philip J. Withers

**Affiliations:** a Wellcome Centre for Cell Matrix Research, The University of Manchester, Oxford Road, Manchester M13 9PL, UK; b Faculty of Biology Medicine and Health, The University of Manchester, Oxford Road, Manchester M13 9PL, UK; c Immunopathology Laboratory, NUPEB, Federal University of Ouro Preto, Campus Universitário Morro do Cruzeiro, 35400-000 Ouro Preto, MG, Brazil; d Henry Royce Institute for Advanced Materials, School of Materials, The University of Manchester, Oxford Road, Manchester M13 9PL, UK

**Keywords:** Ultramicrotomy, Multiscale imaging, Pre-screening, 3View SBF-SEM, IMOD

## Abstract

Transmission electron microscopy (TEM) and scanning electron microscopy (SEM) can provide unrivalled high-resolution images of specific features and volumes of interest. However, the regions interrogated are typically very small, and sample preparation is both time-consuming and destructive. Here we consider how prior X-ray micro-computed tomography (microCT) presents an opportunity to increase the efficiency of electron microscopy in biology. We demonstrate how it can be used to; select the most promising samples and target site-specific locations; provide a wider context of the location being interrogated (multiscale correlative imaging); guide sample preparation and 3D imaging schemes; as well as quantify the effects of destructive sample preparation and staining procedures. We present a workflow utilising open source software in which microCT can be used either broadly, or precisely, to experimentally steer and inform subsequent electron microscopy studies. As automated sample registration procedures are developed to enable correlative microscopy, experimental steering by prior CT could be beneficially routinely incorporated into many experimental workflows.

## Highlights

• Using microCT pre-scans to accurately steer serial block face SEM.
• High throughput screening and mapping samples to reduce time hunting for features of interest.
• Using microCT to optimise specimen preparation and staining.
• Using microCT to guide site-specific TEM sample preparation.

## 1 Introduction

Transmission (TEM) and scanning (SEM) electron microscopy techniques provide high-resolution images across a range of natural sciences. Through destructive serial sectioning, stacks of 2D images can be collected and aligned in sequence to build up three dimensional (3D) volume images [1], [2]. Typically, 3D serial block-face scanning electron microscopic (SBF-SEM) methods involving focused ion beam (FIB) milling [3] or mechanical sectioning [4] are used to provide high resolution information on very small regions of material, and the volumes interrogated by ultramicrotomy and FIB serial sectioning are typically around (1 mm)^3^ and (0.03 mm)^3^, respectively [5]. Therefore, there is a requirement to be able to select the region of interest (ROI) for study with a high degree of site specificity.

It is possible to use information gleaned from inspecting the surface of the sample prior to serial block-face imaging to provide contextual information about the region selected for detailed investigation, thus increasing the chances that the region selected will contain the features of interest prior to embarking on time consuming slicing and imaging workflows. Despite the fact that the volumes interrogated by ion beam methods have been increased somewhat by the advent of plasma FIB microscopes [5] to around (0.3 mm)^3^_,_ capturing the feature of interest is still challenging when the internal morphology of a sample is not known.

In this paper we examine how non-destructive X-ray micro computed tomography (microCT) can be incorporated into multiscale correlative microscopy workflows to ensure high efficiency and quality of electron microscopy. MicroCT is well suited to the imaging of samples of many millimetre dimensions at resolutions approaching a micron and some nanoCT instruments can image sub-millimeter samples at 50 nm resolution [6]. As a non-destructive method, X-ray CT thus provides an opportunity to establish a map of the sample prior to more detailed, guided investigation by electron microscopy methods [7], [8].

In the current work we examine the experimental issues associated with developing a simple correlative workflow. Our approach utilises the freely available and open-source software package IMOD [9] although other software, such as ImageJ/Fiji [10] could be used following the same principles. This workflow is then applied to a number of demonstrator case studies where prior microCT could be a useful preliminary step, namely:

• *For high-throughput screening of multiple samples;* in our case to assess staining and fixation quality in samples of heart and enteric fat tissue prior to the beginning of serial sectioning.
• *To steer 3D serial sectioning workflows:* in our case ensure features (Purkinje fibres in a rabbit heart) are tracked and captured without inefficient wider sampling or, conversely, partially clipping.
• *To identify samples containing specific rare features:* in our case cell clusters in alginate gels.
• *To locate specific features for detailed site-specific investigation:* in our case the head of a whipworm buried within the gut lining of a mouse.

Other potential advantages include using microCT to first generate a coarser scale overview of the sample in order to put into context the regions of interest (ROIs) from which higher resolution electron microscopy images are acquired (multiscale correlative imaging), to orient the sample to optimise subsequent sectioning/excision, or to quantify levels of shrinkage/damage to the sample during sample preparation (fixing, staining or slicing) [11].

Finally we consider future refinements, such as automated sample registration procedures, that would enable correlative imaging or experimental steering by prior CT to be routinely incorporated into experimental workflows.

## 2 Methods

### 2.1 Experimental workflow

**Fig. 1:**
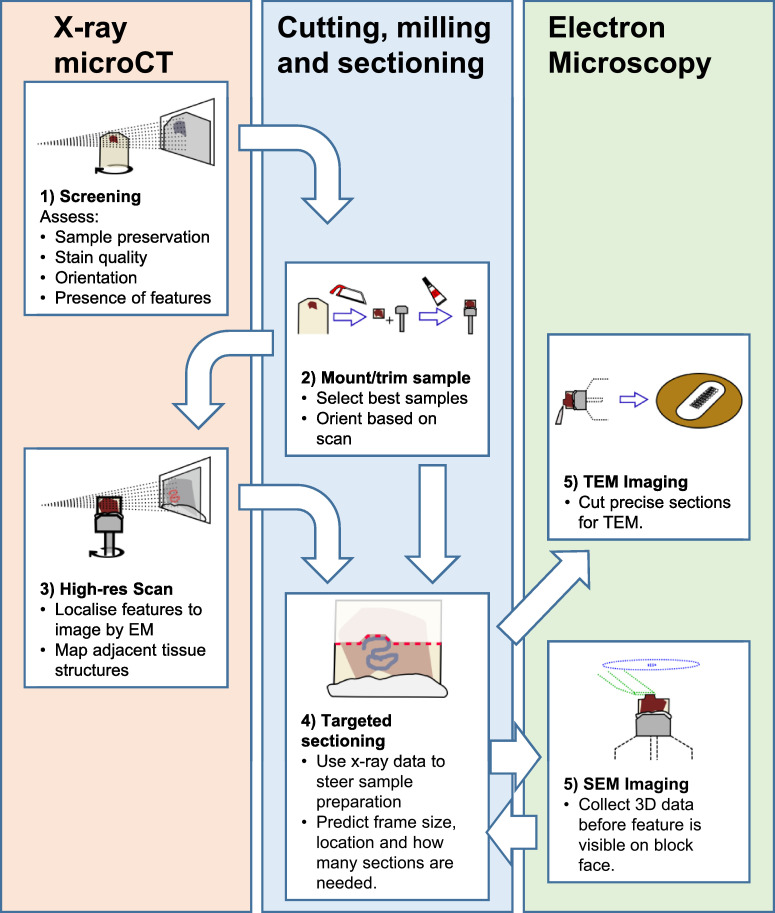
Flow chart for preparing a sample for EM imaging guided by microCT.

While every experimental workflow is different, the main steps for microCT steering of serial block-face SEM (SBF-SEM) imaging or site specific TEM are broadly the same, as illustrated in Fig. 1. Each step is considered in turn below.

#### 2.1.1 MicroCT screening

After samples have been stained and embedded (see Section 2.2.2) they are pre-screened by laboratory CT. This reduces the risk of expending a great deal of time, or expense, trimming and imaging samples which lack relevant biological features. For example, by quickly scanning a range of batch-prepared biopsies, it is possible to pick the best samples for further processing, thereby eliminating unfruitful future imaging acquisitions. This is especially relevant for studies aiming to view rare features, which may be distributed heterogeneously throughout biopsies (for examples see Sections 3.3 and 3.4). Additionally, once a sample of interest has been selected, an overview of the internal morphology also prevents accidental trimming of important regions. While assessing the screening data it is also possible to examine quality and evenness of sample preparation (see Section 3.1) as well the orientation of the feature of interest from the viewpoint of establishing the best sectioning plane. For screening scans, a high-throughput may be more important than maximising resolution, and acquisition settings can be adjusted to collect the data as quickly as possible, for instance by reducing the number of projections, binning pixels on the detector, as well as using short exposure times. Imaging a number of samples in a single field of view, or using a carousel to load scan samples in a sequence are both ways of further increasing throughput of scans.

#### 2.1.2 Mount/trim sample

In the light of the information gathered from the CT scan, one can down-select the most promising samples. For each of these it is possible to orient the sample to optimise subsequent sectioning/excision as well as to aggressively trim the sample to minimise milling/sectioning times. Often this is done fairly coarsely by hand using a razor blade. If it is not the case already, the sample can then be attached to a pin or some other sample stub. This can then be transported directly to the SEM for block-face imaging or sectioned for TEM examination. In cases where reorientation has occurred, and/or a more precise location of a region of interest is required relative to the sample stub before trimming can occur, further CT scanning may be necessary.

#### 2.1.3 High-resolution CT scan

With the sample mounted onto a pin it is now possible to scan at higher resolution in order to more precisely register the location of the features of interest and thereby steer the sample preparation. It is expedient to accommodate the sample within the CT system using the pin/stub which means that the subsequent (horizontal) tomographic slices are oriented normal to the pin. This is especially advantageous for serial block-face SEM because this is parallel to the sectioning plane of the diamond knife. The high-resolution tomographic images can be used as a map that will reveal what lies beneath the surface when the thin sections are removed.

#### 2.1.4 Site specific milling

In order to target a specific region within the sample an ultramicrotome is used to mill a face perpendicular to the pin axis. By controlling the depth of milling and comparing BSEM images of the block-face to the virtual slices in the CT pre-scan/high-res scan it is possible to identify the desired imaging plane. For high-resolution EM imaging the block-face must be accurately trimmed to a size that is compatible with ultra-thin sectioning (<70 nm slices). To trim the block-face with sufficient accuracy, the SEM beam may be used to burn fiducial markers onto the surface of the block; the markers can be positioned at points where the ROI box crosses features that are easily identified in the SEM image (see supplementary information). Once markers have been applied, the sample can be further hand-trimmed under a dissecting light microscope. Using this method it is possible to accurately trim a sample face to within 50–100 µm of the sample X and Y, and to accurately trim the Z depth to within a micron.

#### 2.1.5 Electron microscope imaging

Once the desired block-face has been exposed and trimmed, electron microscopic imaging can begin. It should be noted that the optimal position of the block-face may vary depending on the modality used; for instance, for TEM imaging the diamond trimming knife should be used to get within a single X-ray slice (approx. 1–2 µm, depending on imaging resolution) of the ROI. Alternatively, for volume imaging using serial block-face, or dual beam SEM machines it is possible to use the X-ray data volume to target imaging a few micrometres before the feature of interest is revealed so that it is captured in its entirety by the serial sectioning process. The X-ray data can be used to determine how deep the SBF-SEM volume should be, as well as the precise location and size of the imaging frame so that it includes all of the tissue of interest. By restricting the imaging to the exact sizes needed it is possible to accurately control the duration of the scan. Establishing an exactly-sized imaging frame of the region of interest for a high-resolution acquisition also improves throughput, because the scan does not need to be extended over a wide search area in the hope of capturing a feature of interest.

### 2.2 Experimental procedures

Each of the case studies below highlights a particular opportunity for experimental steering by microCT through the examination of a real world sample. The samples are all biological in nature and have been prepared with the same staining and embedding protocol. There are a number of methods that are common to all of the samples which are as follows:

#### 2.2.1 Experimental animals

All procedures involving animals were carried out in accordance with the Animals for Scientific Procedures Act, under the authorities of home office licenses. For the adipose work (Section 3.1), murine gut adipose was collected and pieces (<1 mm^3^) were placed directly into primary fixative. For the heart tissue (Section 3.2), a rabbit was euthanized and the heart was quickly removed. The specific regions around the pacemaker cells were carefully dissected and the small pieces (<1 mm^3^) were placed directly into vials of primary fixative. For the cell culture work (Section 3.3), primary murine mammary epithelial cells were cultured in an alginate based hydrogel [12]. The cells were fixed by replacing the tissue culture media with primary fixative. For the work on *Trichuris muris* (Section 3.4), SCID mice were infected by oral gavage with 200 T*. muris* eggs, and euthanised 42 days after infection. Regions of the caecum thought to contain worms were cut into 1 mm^3^ and placed into primary fixative.

#### 2.2.2 Staining, fixing, and embedding

All samples were prepared according to a standard SBF-SEM protocol [13]. Sample fragments were fixed in 2.5% Glutaraldehyde, 4% PFA in 0.1 M HEPES pH 7.2 before being stained in 1% Osmium tetroxide, 1.5% potassium ferrocyanide in 0.1 M HEPES, followed by 1% aqueous filtered Thiocarbohydrazide. After washing, the fragments were incubated in 1% aqueous OsO_4_, 1% aqueous Uranyl acetate, and finally in Walton's lead aspartate [14]. Samples were dehydrated in graded ethanol and infiltrated with TAAB 812 Hard resin, which was cured in an oven at 60 °C for between 24 and 48 h.

#### 2.2.3 MicroCT imaging

For the purpose of this study all CT imaging was carried out on a Zeiss Versa 520 X-ray microscope using a 4x objective lens. Due to the high stain content the images gave good contrast at 60 kV accelerating voltage with maximum power (5 W). A variety of imaging regimes were used depending on the resolution of information required. The scan parameters are detailed in Table 1.

**Table 1:** X-ray scan parameters used to collect the data sets presented in this paper.

Sample	Scan type	Image size (*X*/*Y*) in pixels	Number of frames	Pixel size (µm)	Frame time (s)	Total scan time (hour:min)
Acini	Screening (1 sample)	1012 (bin2)	72	2.152	8	00:14
Heart	Screening (1 sample)	1012 (bin2)	401	2.966	2.5	00:21
Adipose	Screening (12 samples)	1024 (bin2)	201	15.11	2	00:21
	High-res. (2 samples)	2028	1601	2.264	8	03:37
*Trichuris* in gut	Screening (1 sample)	1012 (bin2)	72	1.773	10	00:16
	High-res. (1 sample)	2028	1601	1.827	20	08:59

CT scans were reconstructed using filtered back-projection. The data were analysed and rendered using Amira 6.4 (Thermo Fischer Scientific). For targeting of EM imaging the MRC file format [15], [16] was used. The contrast was inverted in IMOD [9] in order to directly compare the 2D slice images with images collected by EM.

#### 2.2.4 Trimming the sample for serial sectioning

In each case coarse manual trimming was first applied to reduce the size of the sample. In order to ensure the sample was cut to the minimum size such that the regions of interest were retained, the X-ray data had to be compared with an image of the sample block face. The X-ray data comprises a series of virtual slices through the volume being imaged. The data can be converted into various formats which can be opened using a number of programs. We used IMOD to view the X-ray slices [9] (http://bio3d.colorado.edu) (Fig. 7), but other software packages such as Fiji may also be used [10]. A box was then defined to enclose each of the underlying regions of interest in the X-ray data. The individual boxes were then copied onto the X-ray section that mapped to the block-face as seen in the SEM. By comparing the position of the underlying ROIs with the SEM block-face image it is possible to work out how much of the sample could be safely trimmed. The depth of the ROI nearest to the block-face plane is used to decide how much resin can be removed with the trimming knife. The sample was then trimmed using a diamond trimming knife on a standard ultra-microtome (Reichert–Jung Ultracut) counting 500 nm thick cuts. Periodically the size of the sample face needed to be reduced in order to reduce strain on the knife. In order to accurately trim the sample a number of fiducial markers were introduced onto the resin surface to guide manual trimming. To do this the sample was placed on the stage of the SEM and fiducial spots introduced by turning the beam to 30 kV and magnification 50 K and then parking the beam to introduce beam damage onto the resin surface for approximately 20 s (this is explained in more detail in the Supplementary information).

#### 2.2.5 Collection of the serial block-face SEM data

The EM data was collected on a 3View ultra-microtome (Gatan, UK) fitted to an FEI Quanta 250 FEG (Thermo Fischer Scientific). The imaging regimes were tailored to the sample being imaged as summarised in Table 2.

**Table 2:** Serial Blockface SEM parameters used to collect the data sets presented in this paper.

Sample	Accelerating voltage (kV)	Chamber pressure (Torr)	Image frame (pixels)	Pixel size (nm)	Dwell time (ms)	Slice thickness (nm)
Acini	3.5	0.28	4000 × 4000	15	5	60
Heart, Purkinje fibre	3.8	0.45	(1) 5000 × 5000	30	3.5	150
			(2) 8192 × 5000			
			(3) 6750 × 5000			
Trichuris, worm head	3.8	0.47	6000 × 7000	13.47	3.3	60

## 3 Results and discussion

### 3.1 High-throughput screening; assessing/optimising the quality of sample preparation

In order to collect good quality 3View images it is important that samples are uniformly fixed, so that ultrastructural features are preserved through subsequent processing. In addition, the *en bloc* staining species employed (e.g., Osmium tetroxide, Uranyl acetate) must penetrate the sample sufficiently such that features of interest have enough contrast in the back-scattered electron micrographs. Conventionally, semi-thin sectioning and light microscopic imaging may be employed to determine staining efficacy. Here we propose the use of X-ray tomography instead. Although the use of X-ray imaging is not suited to assessing the quality of resin infiltration, nor the formation of fine precipitates, it may be advantageous to use over a sectioning approach, as it avoids cutting away potentially important parts of the sample, and has the potential to achieve a higher throughput by screening many samples at the same time in batches using a quick scan.

**Fig. 2:**
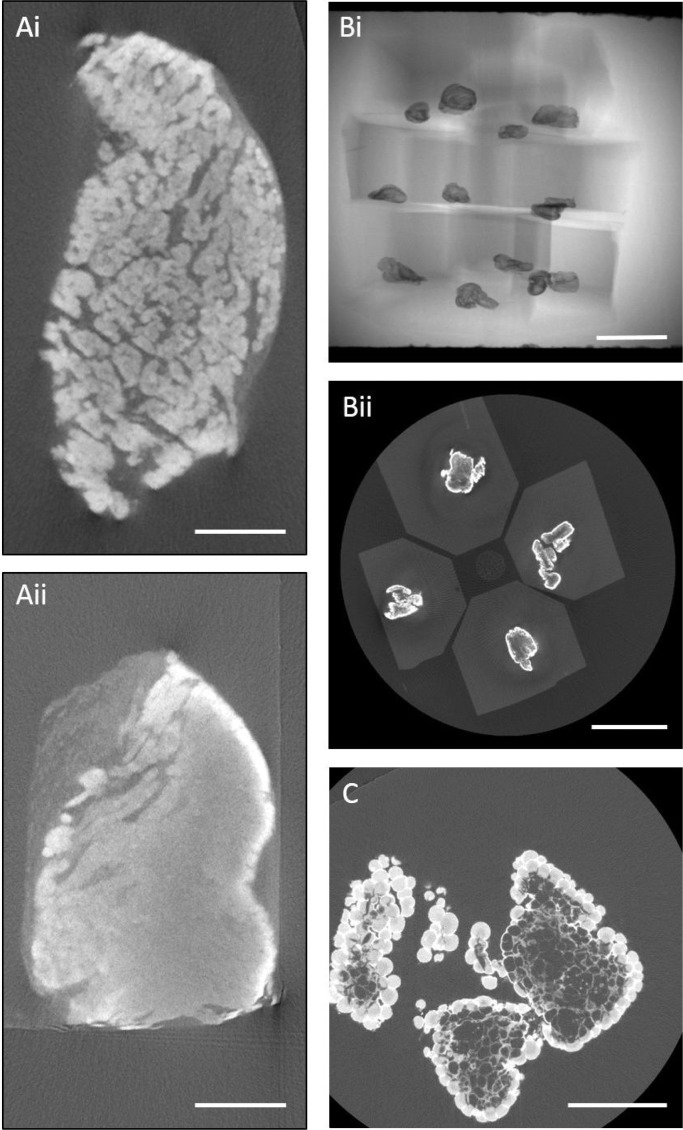
*Quick assessment of stain penetration can be supplemented by high throughput screening of samples.* Using microCT, stain quality and fixative penetration can be assessed before beginning the time-consuming trimming and sectioning. (**A**) microCT virtual slices of two murine heart samples stained for SBF-SEM can be compared. The top sample (**Ai**) shows good stain penetration seen as the uniform absorption across the sample with detail seen in the separation of the individual fibers, while the sample on the bottom (**Aii**) shows poor stain penetration with more X-ray absorption around the periphery and no detail in the centre. Scale bar 25 µm. (**B**) Radiograph (**Bi**) and virtual slice (**Bii**) of a high-throughput scan of 12 resin-embedded samples of fat. Scale bars 3 mm (**C**) In a high-resolution scan of a single piece of fat, a clear front of fixative penetration is perceptible. The lipids in the interior of the sample have been extracted by dehydration in acetone leaving only the matrix and cell cytoplasm. Scale bar 500 µm.

Here we consider two case studies in which pre-screening SBF-SEM samples by microCT is used to non-destructively assess the quality of sample preparation, namely samples of murine cardiac muscle and fat from the gut. In the former case insufficient stain penetration is diagnosed by a clear front of increased contrast towards the outer edges of the sample (Fig. 2 Ai, ii). The top sample (Ai) shows good stain penetration seen as the uniform absorption across the sample, while the sample on the bottom (Aii) shows poor stain penetration with more X-ray absorption around the periphery. In the second example, many samples are grouped together and imaged in a single run (Fig. 2(B)). In this way the status of multiple samples could be checked at the same time increasing the efficiency (the total time to screen 12 samples was 7 min). This high throughput screening process shows the failure of the fixative (Osmium tetroxide, OsO_4_) to sufficiently penetrate the sample leading to partial fixation of the lipids. This has resulted in the extraction of fat cells from the centre of the sample during dehydration in acetone, leaving behind empty portions of extracellular matrix (Fig. 2(C)). This evidence of insufficient penetration of reagents into the tissue facilitates the improvement of the preparation method prior to costly SBF-SEM, either by using longer staining times to increase penetration [17], [18], or by reducing the size of the tissue samples to attain more uniform staining. Alternatively, in some circumstances the outer regions into which stain has penetrated well could serve as a ROI for further electron microscopic examination.

### 3.2 Planning serial block-face imaging campaigns to efficiently track features: Purkinje fibres in a rabbit heart

In many cases there are morphological, resolution and cost efficiency arguments for orienting the region of interest and carefully planning the extent of sectioned volume before carrying out sectioning. For example, by scoping the region of interest and orienting the sample it is possible to track features in the most time efficient manner while ensuring the region of interest doesn't leave the field of view as sectioning progresses. Secondly, serial sectioning results in anisotropic resolution of the final volume data, because the sectioning thickness (which determines the axial resolution) is, in most cases, significantly larger than the pixel size (which determines the lateral resolution) within each BSEM image [2]. Because of this inherent anisotropic resolution, the orientation of samples should be chosen such that features of greatest interest are intersected optimally by the plane of imaging and can be viewed at the superior lateral resolution.

**Fig. 3:**
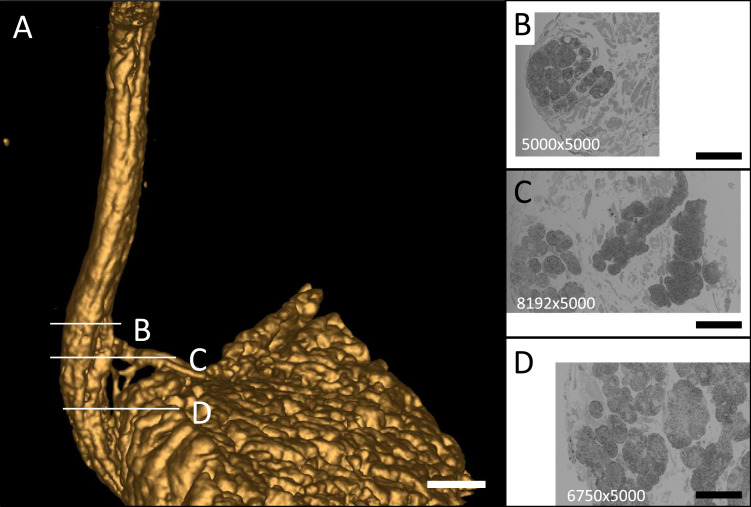
*Adjusting SBF-SEM imaging to optimise the acquisition time while retaining the features of interest in the volume.* (**A**) Surface rendering of an X-ray tomogram (acquired on a Zeiss Versa 520) showing the trajectory of the Purkinje Fibre tracking down to cardiac tissue. Using the microCT data it was possible to orient and define the sectioning scheme so as to image the region where a fibre splits from the main bundle to connect to the cardiac wall. (**B–D**) example SEM images collected at different points along the branch as part of an SBF-SEM volume. Scale bars represent 50 μm.

In our case the aim was to examine the cellular morphology of the pacemaker tissue and in particular examining the ultrastructure of the sinoatrial node (SAN) cells and Purkinje fibres (PFs) of the rabbit conduction system. In this case X-ray steering enables the volume to be sectioned to be optimised, without danger of accidentally cropping part of the ROI. From this viewpoint it is desirable for the sample to be oriented such that the fibre runs in the z-orientation so that the 3View field of view can be minimised using a square frame. The X-ray data in Fig. 3 shows that at a depth of around 290 μm the fibre turns to run near parallel to the image frame. With this prior information we were able to change the 3view sectioning scheme from a square image frame (Fig. 3(B)) to a larger one at the appropriate point to include the whole fibre (Fig. 3(C)). Once we had passed this point we were able to continue imaging with the standard rectangle frame (Fig. 3(D)). Given that the image acquisition time is based on the total number of pixels per frame we were able to optimise the acquisition sequence and thus acquisition time beforehand without any risk of losing the features of interest from the frame.

### 3.3 Locating hidden or rare features: cell clusters in alginate gels

Blind SBF-SEM is a very unsatisfactory and inefficient way of locating and imaging rare features. In such cases pre-screening by microCT has clear benefits. Here we consider a 3D cell alginate based cell culture model to examine the role of the extra-cellular matrix environment in the control of cell proliferation and programmed cell death in a model of mammalian milk production [12], [19]. In such a sample, a range of fluorescent microscope techniques might be used in order to understand the proteins that are being produced and their localisation. However fluorescent microscopy necessitates the use of specific stains and means that the precise environment cannot be examined. By utilising a non-specific stain and the high-resolution of electron microscopy, the cell-matrix interactions across the surface of the acini as well as the polarisation of the cells between the external matrix and the milk filled lumen, can be examined. However, the acini are randomly and sparsely spread throughout the matrix. Pre-scanning by microCT obviates the need to randomly sample large areas at low magnification in the hope of imaging the acini, or to target cells that are partially exposed on the surface of the block, knowing that we could only collect the portion of the cluster that is left beneath the surface.

**Fig. 4:**
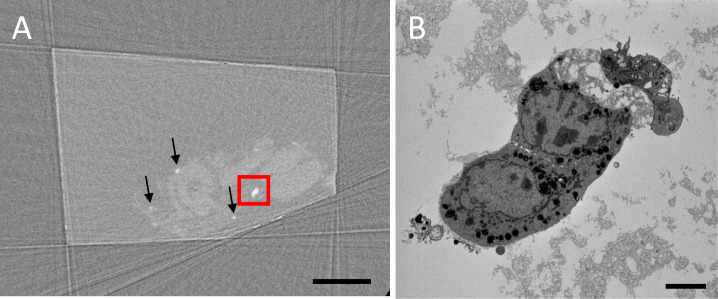
*Rare features can be imaged in their entirety.***(A**) Using fast (72 projection) X-ray tomogram pre-scanning it is possible to locate rare cell clusters grown in an alginate matrix. The short time and limited projections obtained are evident by the streak artefacts present on the virtual slice. However, resolution and contrast is sufficient to correctly obtain the locations of the cell clusters (arrows and within red box). (**B**) Example back-scattered SEM image from the same region boxed in red in **A**. (For interpretation of the references to colour in this figure legend, the reader is referred to the web version of this article.)

A large microCT volume (7.3 mm^3^) was acquired prior to destructive SBF-SEM to locate whole acinus (Fig. 4(A)). This screening was undertaken quickly acquiring just 72 radiographs (projections). While this leads to streak artefacts and noise in the X-ray image the resolution and contrast is sufficient to obtain the locations of the acini. With the location identified from the microCT, the ROI was excised and SBF-SEM undertaken confident in the knowledge that the smaller volume was known to contain the whole acinus (Fig. 4(B)).

### 3.4 Site specific imaging: locating the head of Trichuris muris within a mouse gut lining

*T. muris* is a commonly used model of the human endoparasite *Trichuris trichiura* (whipworm), which is responsible for Trichuriasis, one of the most common neglected tropical diseases in the world [20]. The *Trichuris* genus is characterised by their unique niche; they live partly embedded within the epithelium of the caecum and proximal colon, in a “tunnel” made by burrowing through adjacent epithelial cells [21], [22]. Capturing the anterior, embedded part (‘head’) of the worm *in situ* is important for EM studies attempting to characterise the formation, maintenance and development of the epithelial tunnel. This is because there are many highly specialised, yet poorly understood surface structures such as the bacillary pores which characterise this region and are most likely important in facilitating the worm's intra-epithelial lifestyle [23]. A better understanding of the host-parasite interaction underpins future control strategies targeting this parasite. *In situ* imaging of anterior structures on *Trichuris* within the epithelial tunnel is challenging because 1) in gut biopsies the orientation of the embedded worm is impossible to determine from external inspection and 2) the bacillary pores, whilst of a consistent appearance on the surface of the worm, are functionally heterogeneous and consist of a variety of cell types distributed beneath the surface [22], [24]. The combination of these factors means that the region of worm embedded for sectioning and its orientation are often determined blindly. Perhaps as a result of this, the functions of many *Trichuris* surface structures remain the subject of speculation (e.g., [25]).

**Fig. 5:**
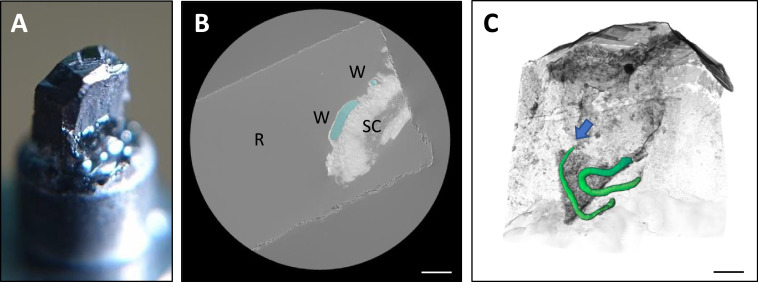
*Examining X-ray data reveals hidden sub-surface features.* (**A**) Photo-micrograph of a mounted 3-view sample coated in gold palladium. (**B**) Virtual slice through X-ray volume showing the presence of a *Trichuris* parasite (W) embedded in the stained cecum tissue (SC). The shape of the resin block (R) can be seen as a white outline caused by X-ray absorption from the gold palladium coating. (**C**) Volume rendering of *T. muris* (green), showing its positioning within the fragment of gut tissue. The anterior region of the worm, which is the region of interest, is marked by a blue arrow. Scale bars 500 µm. (For interpretation of the references to colour in this figure legend, the reader is referred to the web version of this article.)

Here the aim was to target the anterior-most region of the worm and hence identify the pathologically relevant elements of worm and host morphology. Consequently, pre-screening microCT was used firstly to identify a sample worthy of investigation (i.e., one containing a worm) and secondly to steer the SBF-SEM in order to target the embedded anterior most region. Within the opaque resin embedded gut sample (Fig. 5(A)), a length of worm was revealed through inspection of the slices (Fig. 5(B)). Moreover, the presence of the anterior region was confirmed, the tip of which was noted as the desired location for the block-face imaging (Fig. 5(C)).

**Fig. 6:**
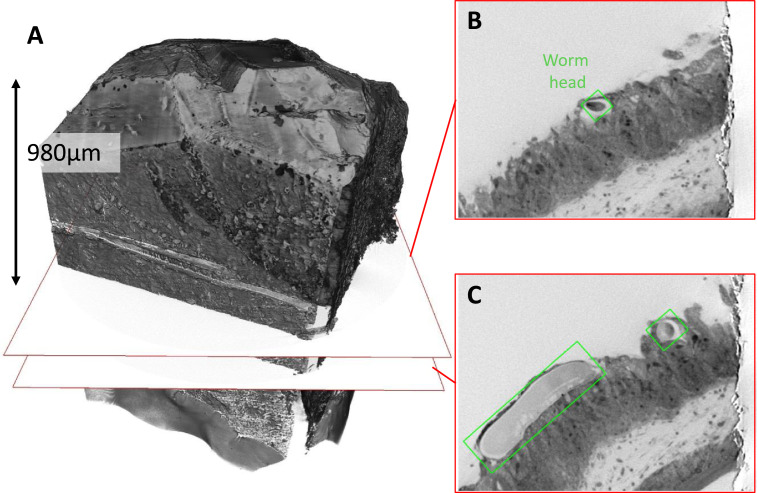
*Identifying regions of interest within the X-ray data set to target subsequent trimming.***(A**) A magnified volume render of the resin sample generated from the X-ray data. (**B**) Virtual XY section showing the worm head buried within the cecum tissue. Analysis of the X-ray tomogram shows that this feature is found 980 μm below the current block-face. (**C**) A virtual XY CT slice showing the same worm head 180 μm deeper into the sample. At this point it is also possible to see part of the body of the whipworm (within the green rectangle, modelled in 3dmod).

**Fig. 7:**
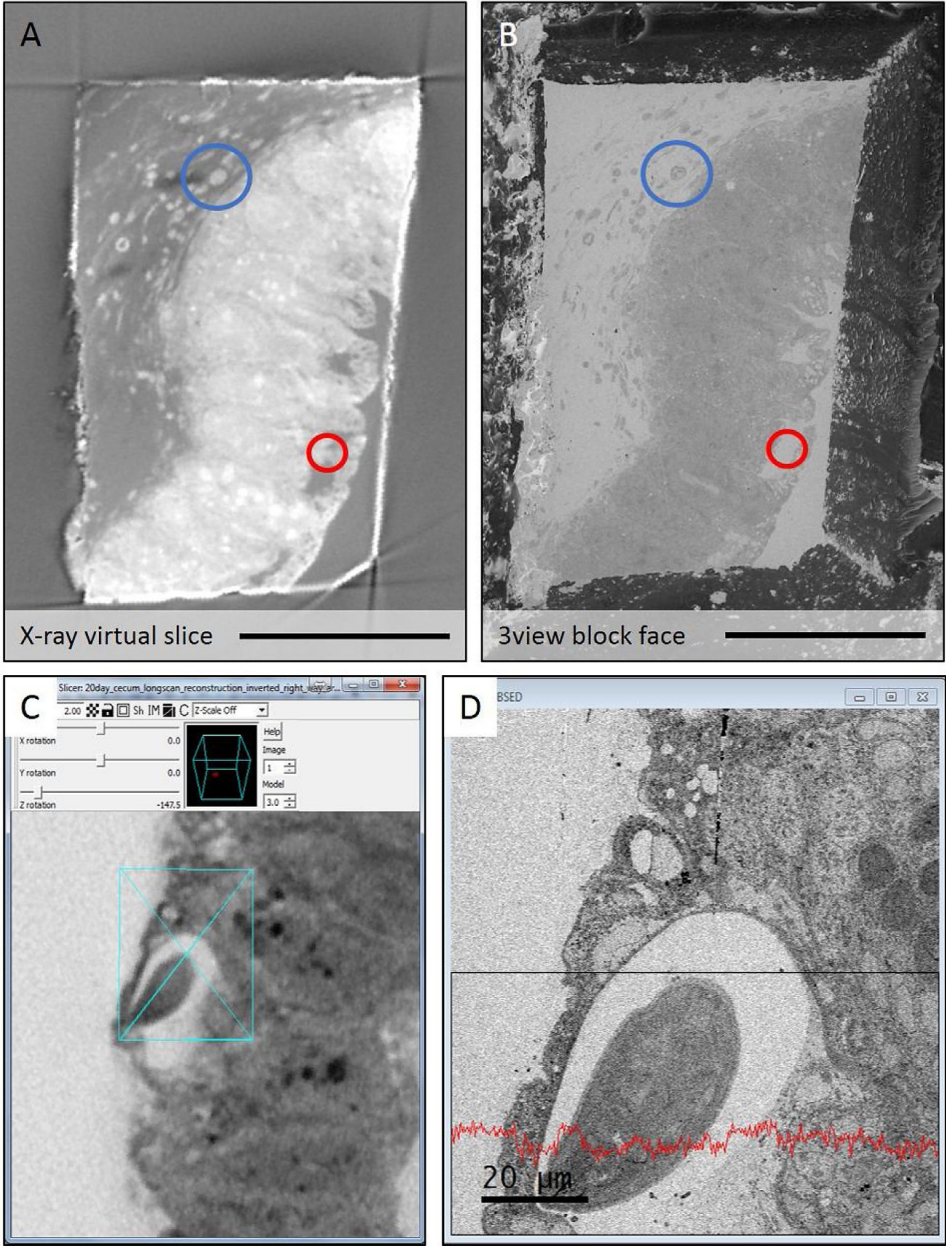
*Side-by-side comparison of a virtual slice from the microCT.* (**A**) Virtual slice from the microCT and back scattered SEM image (**B**) collected from the block surface revealed by the 3View. Gross morphology such as small capillaries (blue, circled) aid in registering the two datasets. Small differences in registration between the images due to slight miss-registration (circled red). (**C**) Virtual section from the microCT of the worm head visualised in IMOD using ‘Slicer’. (**D**) SBF-SEM image of the same region collected live in Digital Micrograph. Videos of the corresponding stacks of the virtual (CT) and real slices (SEM) side by side are included in the supplementary material. (For interpretation of the references to colour in this figure legend, the reader is referred to the web version of this article.)

The microCT reveals that the head lay approximately 980 μm from the top of the sample (Fig. 6). As well as facilitating the optimal positioning of the block-face before beginning a 3View run, X-ray data can be used to determine the position of features of interest at the end of the data acquisition. In practice small differences in the calibration and alignment between the X-ray and SEM systems mean that there was a slight dis-registry between the two coordinate frames, however the similar appearance of gross morphological features between the two data sets confirms that the SBF-SEM imaging frame was appropriately located (Fig. 7). By using IMOD, it was possible to centre the SEM image acquisition and use CT steering to manage the whole sectioning and image acquisition process.

**Fig. 8:**
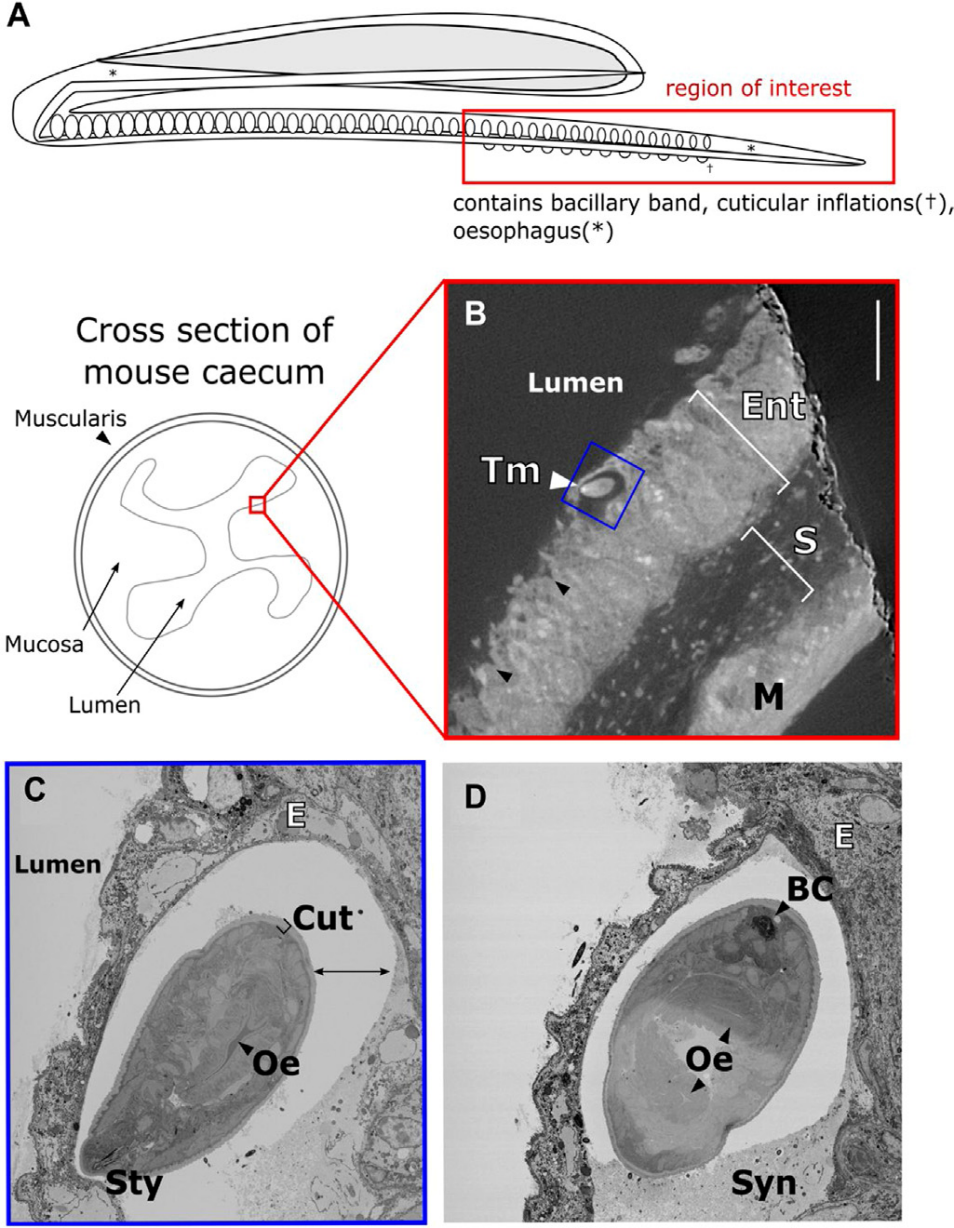
*The correlative workflow allows location and identification of key elements of Trichuris muris morphology.* (**A**) Schematic of *T. muris* showing the area of worm relevant to the current study. (**B**) X-ray virtual slice of stained, resin-embedded tissue showing the tip of the *T. muris* head (Tm), the enterocytes consisting the mucosa (Ent), sub-mucosa (S) and muscularis (M). Scale bar 100 µm. (**C+D**) Backscatter electron micrographs of the block-face showing the morphology of the tip of the worm head (**C**) and the body at a point 300 microns further into the block (**D**) in more detail, including the Stylet (Sty), oesophagus (Oe) and cuticle (Cut, square bracket), as well as the bacillary cell (BC) and syncytium which surrounds the worm (Syn). The worm is surrounded by epithelial cells (E). There is also a gap between the worm and the surrounding syncytium marked by the double-headed arrow, which is likely a shrinkage artefact.

As a result of X-ray steered SBF-SEM imaging, key aspects of the whipworm morphology have been identified (Fig. 8). In the microCT slices, mucosal morphology is evident, including the layers composing the gut wall (muscularis, submucosa, mucosa), as well as features such as the crypts of Lieberkuhn (Fig. 8(B)). The structure of the mucosa itself is important as it contains goblet cells which secrete mucus and are important in the control of *T. muris* infection [26]. Subsequent higher resolution SBF-SEM images (Fig. 8C+D) revealed finer structural elements of the worm itself, including the oesophagus, cuticle, stylet, and bacillary cells. The syncytium which surrounds the worm as it burrows through the host gut lining is also clearly visible, although empty resin between the syncytium and worm suggests that sample shrinkage has occurred.

## 4 Comments on the workflow

A wide range of correlative imaging approaches which draw together diverse imaging and mapping modalities to establish a richer picture of materials are gaining traction [27]. Within many of these, X-ray micro-computed tomography features as a first step, to provide temporal (time-lapse) information, to combine different types of data collected using different modalities, or as a part of multiscale studies. Here, we have looked at the use of microCT a first step in biological studies, with a particular focus on its use for experimental steering. We have presented a complete workflow which is achievable using open source software, and have provided a number of case studies that illustrate the potential benefits, but also some of the experimental issues that must be tackled. During the course of these experiments several areas of improvement and refinement were identified, achievable either in the immediate future or over longer timescales, which are discussed in the following paragraphs.

### 4.1 Extension of the workflow to other EM techniques

Whilst we have described how X-ray screening of samples can be utilised to increase efficacy of SBF-SEM, other EM modalities would also benefit from similar pre-screening and experimental steering. For instance, CT pre-screening allows site-specific excision of TEM slices so as to capture specific features within the sample. Further 50 nm serial TEM sectioning campaigns can be planned to collect higher-resolution 3D data than is possible by SBF-SEM. An alternative, higher throughput approach would be to collect a series of thick (300 nm) sections to cover the region of interest. Here serial section electron tomography can be used to generate a low magnification volume covering the ROI [28]. The sections can then be interrogated at higher magnification once the ROI has been accurately identified.

### 4.2 Automated registration of datasets

For a variety of reasons, the accurate and automated registration of images between imaging modalities is of increasing interest to researchers utilising correlative approaches. For instance in Correlative Light and Electron Microscopy (CLEM), the registration of fluorescence data is important for matching the detailed ultrastructural information of EM with patterns of gene expression and/or protein localisation [29]. In the workflow we describe above, registration of BSEM images with equivalent microCT slices has been carried out manually. However, there are a variety of ways in which future application of automated imaging registration of EM and X-ray data could increase the efficiency of data collection and visualisation. For instance registration of the datasets may not only be useful in validating the correct interpretation of features in both datasets, but also in quantifying any artefacts introduced into the morphology due to the action of the microtome [11].

From a data interpretation point of view, superimposing volume renderings and slices of both SBF-SEM and microCT data aids in putting ultrastructure within the context of the surrounding 3D tissue morphology, thus providing greater clarity to readers interested in the exact position of the imaging window. Automatic registration of CT and BSEM images before the commencement of data collection, that is, during the trimming stage may also assist in speeding up critical elements of the workflow. For instance, when trimming the block-face to achieve the desired starting point, automated registration of the block-face with slices of the CT data could facilitate quick quantification of changes in orientation introduced by the investigator.

### 4.3 Automation of block-face preparation

Whilst we have discussed how automated image registration may allow the streamlining of the existing workflow as presented above, perhaps the greatest savings of time and effort may be gained from the automation of the block trimming itself. For instance, in correlative microCT and FIB-SEM, X-ray data can be used to guide subsequent automated ion beam milling and acquisition of EM volumes [30]. In the workflow presented in this paper, the X-ray data has been used to inform manual trimming of the sample resin using razor blades and microtomy. However, coarse trimming by eye takes time and effort, and whilst precise trimming by microtomy is necessary to achieve the correct depth of the imaging frame, other trimming approaches may increase the ease of the trimming process. One easy way to increase accuracy and efficiency of block-face trimming may be to include fiducial markers on the SEM stub/pin prior to microCT scanning, in order that the block-face may be trimmed with quick reference to clear landmarks [8]. However using X-ray data as a guide for automated trimming is the ultimate goal for high-throughput and accuracy of block-face localisation. As mentioned, automated FIB milling is one approach to achieving this goal, however, this may be too slow a process for resin blocks larger than a few millimetres; instead laser cutting, or even high-precision Computer Numerical Control (CNC) machining may be best suited to high-throughput applications, or applications where the resin blocks are above several mm^3^ in volume. Ultimately, it seems likely that continuing development of correlative imaging will exploit automated sample excision and preparation.

### 4.4 Logistical considerations associated with the workflow

In addition to the experimental aspects, which we have demonstrated and discussed in detail, those wishing to employ the correlative approach outlined above should be aware of logistical issues associated with accessing and exploiting microCT instruments. The costs of using instrumentation are an important consideration, and as with any modality, experiments must be planned around availability of the instruments themselves. In our experience, survey scans can take of the order of minutes and the data can be retrieved and utilised to its full extent almost immediately following a scan, as virtually no post-processing of the virtual slices is required. Once the data are retrieved, the availability of computational power may affect the speed of data visualisation and manipulation; however, facilities which already have 3D imaging instruments (e.g., an SEM with 3View) should already be sufficiently well equipped in this area.

## 5 Conclusions

Through a series of diverse case studies we have shown that experimental steering by microCT can significantly reduce the time and increase the cost effectiveness and productivity of 3D and 2D electron microscopy. We were able to use a simple workflow based on open-source software to screen samples and identify those which are fixed and stained appropriately, and contain regions of interest before the beginning of the serial sectioning process. In the case of the Purkinje fibres, microCT was useful in orienting the sample to achieve the best resolution and to track the fibres through an appropriately shaped volume that minimised unnecessary data collection. The method is able to pre-screen many samples quickly and efficiently both to find the best ones for detailed SEM examination but also to ensure that the staining procedures are optimised for best contrast. One of the key benefits of pre-screening by CT is the ability to precisely locate and examine specific features. In our case by accurately registering high resolution CT images with the proposed serial sectioning process we were able to target the buried anterior region of the parasitic whipworm *Trichuris muris* within the a gut biopsy of its murine host, as well as alginate cell clusters embedded in a matrix. Other potential uses include the quantification of the effects of specimen preparation on the integrity of the sample in terms of quantifying non-uniform deformation (uneven shrinkage) or damage during induced fixing, staining or sectioning.

Finally we see advances in correlative imaging, the development of higher precision sample trimming and excision capabilities, the increased connectivity between instruments and automated image registration schemes having significant benefits in terms of the ease with which microCT driven experimental steering can be applied. Once this becomes more routine we envisage the application of pre-EM microCT to a wide range of problems.

## Declarations of interest

None.

## Author contributions

TS Conceived and designed the methodology, collected data and wrote the paper and together with PJW developed X-ray steering concept.

JDBO Performed analysis, improved protocols and wrote the paper, developed concept.

CMC Contributed samples, collected data.

JB Setup x-ray tomography and helped design of multiple scanning regimes.

KE Contributed to analysis of data.

RG Funded the research.

PJW Funded the research, wrote the paper and developed the X-ray steering concept.

All authors have approved the final article.

## Appendix Supplementary materials

Image, application 1

Image, video 1

## References

[1] Zankel A., Wagner J., Poelt P. (2014). Serial sectioning methods for 3D investigations in materials science. Micron.

[2] Peddie C.J., Collinson L.M. (2014). Exploring the third dimension: volume electron microscopy comes of age. Micron.

[3] Heymann J.A.W., Hayles M., Gestmann I., Giannuzzi L.A., Lich B., Subramaniam S. (2006). Site-specific 3D imaging of cells and tissues with a dual beam microscope. J. Struct. Biol..

[4] Denk W., Horstmann H. (2004). Serial block-face scanning electron microscopy to reconstruct three-dimensional tissue nanostructure. PLoS Biol.

[5] Daly M., Burke M.G., Contreras L., Winiarski B., Withers P.J., Burnett T.L., Kelley R., Gholinia A. (2015). Large volume serial section tomography by Xe Plasma FIB dual beam microscopy. Ultramicroscopy.

[6] Maire E., Withers P.J. (2014). Quantitative X-ray tomography. Int. Mater. Rev..

[7] Sengle G., Tufa S.F., Sakai L.Y., Zulliger M.A., Keene D.R. (2013). A correlative method for imaging identical regions of samples by micro-CT, light microscopy, and electron microscopy: imaging adipose tissue in a model system. J. Histochem. Cytochem..

[8] Bushong E.A., Johnson D.D., Kim K.Y., Terada M., Hatori M., Peltier S.T., Panda S., Merkle A., Ellisman M.H. (2015). X-ray microscopy as an approach to increasing accuracy and efficiency of serial block-face imaging for correlated light and electron microscopy of biological specimens. Microsc. Microanal..

[9] Kremer J.R., Mastronarde D.N., McIntosh J.R. (1996). Computer visualization of three-dimensional image data using IMOD. J. Struct. Biol..

[10] Schindelin J., Arganda-Carreras I., Frise E., Kaynig V., Longair M., Pietzsch T., Preibisch S., Rueden C., Saalfeld S., Schmid B., Tinevez J.Y., White D.J., Hartenstein V., Eliceiri K., Tomancak P., Cardona A. (2012). Fiji: an open-source platform for biological-image analysis. Nat. Methods..

[11] Albers J., Markus M.A., Alves F., Dullin C. (2018). X-ray based virtual histology allows guided sectioning of heavy ion stained murine lungs for histological analysis. Sci. Rep..

[12] Wood A.M., Sun H., Williams J., Brennan K.R., Gilmore A.P., Streuli C.H. (2018). Three-dimensional breast culture models: new culture models for analyzing breast development and function. New culture models for analyzing breast development and function. Organoids and Mini-Organs.

[13] Lev-Ram V., Shu X., Ellisman M., Bushong E., Deerinck T., Tsien R. (2010). Enhancing serial block-face scanning electron microscopy to enable high resolution 3-D nanohistology of cells and tissues. Microsc. Microanal..

[14] Walton J. (1979). Lead asparate, an en bloc contrast stain particularly useful for ultrastructural enzymology. J. Histochem. Cytochem..

[15] Crowther R.A., Henderson R., Smith J.M. (1996). MRC image processing programs. J. Struct. Biol..

[16] Cheng A., Henderson R., Mastronarde D., Ludtke S.J., Schoenmakers R.H.M., Short J., Marabini R., Dallakyan S., Agard D., Winn M. (2015). MRC2014: extensions to the MRC format header for electron cryo-microscopy and tomography. J. Struct. Biol..

[17] Aslanidi O.V, Nikolaidou T., Zhao J., Smaill B.H., Gilbert S.H., Holden A.V, Lowe T., Withers P.J., Stephenson R.S., Jarvis J.C., Hancox J.C., Boyett M.R., Zhang H. (2013). Application of micro-computed tomography with iodine staining to cardiac imaging, segmentation, and computational model development. IEEE Trans. Med. Imaging.

[18] Mikula S., Denk W. (2015). High-resolution whole-brain staining for electron microscopic circuit reconstruction. Nat. Methods.

[19] Wood A., Streuli C.H., Foster F., Walker S., Brennan K., Gilmore A.P., Owens T. (2016). Oncogenic activation of FAK drives apoptosis suppression in a 3D-culture model of breast cancer initiation. Oncotarget.

[20] Pullan R.L., Smith J.L., Jasrasaria R., Brooker S.J. (2014). Global numbers of infection and disease burden of soil transmitted helminth infections in 2010. Parasites Vectors.

[21] Lee T.D.G., Wright K.A. (2008). The morphology of the attachment and probable feeding site of the nematode Trichuris muris (Schrank, 1788) Hall, 1916. Can. J. Zool..

[22] Tilney L.G., Connelly P.S., Guild G.M., Vranich K.A., Artis D. (2005). Adaptation of a nematode parasite to living within the mammalian epithelium. J. Exp. Zool. Part A Comp. Exp. Biol..

[23] Hansen T.V.A., Hansen M., Nejsum P., Mejer H., Denwood M., Thamsborg S.M. (2016). Glucose absorption by the bacillary band of trichuris muris. PLoS Negl. Trop. Dis..

[24] Wright K.A., Chan J. (1973). Sense receptors in the bacillary band of trichuroid nematodes. Tissue Cell.

[25] Hüttemann M., Schmahl G., Mehlhorn H. (2007). Light and electron microscopic studies on two nematodes, Angiostrongylus cantonensis and Trichuris muris, differing in their mode of nutrition. Parasitol. Res..

[26] Wynn T.A., Dickey B.F., Hasnain S.Z., Thornton D.J., Wilson M.S., Gallagher A.L., Evans C.M., Kindrachuk K.N., Roy M., Grencis R.K., Barron L. (2011). Muc5ac: a critical component mediating the rejection of enteric nematodes. J. Exp. Med..

[27] Burnett T.L., McDonald S.A., Gholinia A., Geurts R., Janus M., Slater T., Haigh S.J., Ornek C., Almuaili F., Engelberg D.L., Thompson G.E., Withers P.J. (2014). Correlative tomography. Sci. Rep..

[28] Lu Y., Humphries S.M., Kadler K.E., Starborg T., Mironov A., Holmes D.F., Kalson N.S. (2013). Nonmuscle myosin II powered transport of newly formed collagen fibrils at the plasma membrane. Proc. Natl. Acad. Sci..

[29] Acosta B.M.T., Bouthemy P., Kervrann C. (2016). A common image representation and a patch-based search for correlative light-electron-microscopy (CLEM) registration. Proceedings of the International Symposium on Biomedical Imaging.

[30] Kelley R., Tuck O.C.G., Pickering E.J., Léonard F., Daly M., Withers P.J., Sherry A.H., Burnett T.L. (2017). A multi-scale correlative investigation of ductile fracture. Acta Mater..

