# Supplementary material for: Experimental steering of electron microscopy studies using prior X-ray computed tomography
Source: Ultramicroscopy. 2019 Jun;201:58–67. doi: 10.1016/j.ultramic.2019.03.002 (PMC6504073; doi:10.1016/j.ultramic.2019.03.002)
Supplement: Supplementary file 1 [file mmc1.docx]

# Accurate sample trimming by marking the surface in a standard SEM

*Inspecting the block-face with the assistance of microCT data*

In order to accurately trim the EM sample using the X-ray data as a guide we have used our 3view system, but any SEM with a BSED detector will do. We installed the standalone version of 3dmod (the visualization part of the IMOD suite of software [1]), but it should be noted that IMOD may be substituted with any other volumetric viewing software which allows visualisation and marking of virtual slices. The standalone version of 3dmod is available from the SerialEM website (bio3d.colorado.edu/SerialEM/. Note: SerialEM is free to use software suite for automated collection of data on a TEM [2], the installation of which is not required in order to use 3dmod; instead follow the link for the standalone 3dmod in the “Other Helpful Materials” section (link confirmed 20/11/2018)). Using 3dmod, we directly compared the images of the freshly polished block-face with the X-ray data (in the form of either MRC stacks or 2D/3D Tifs).


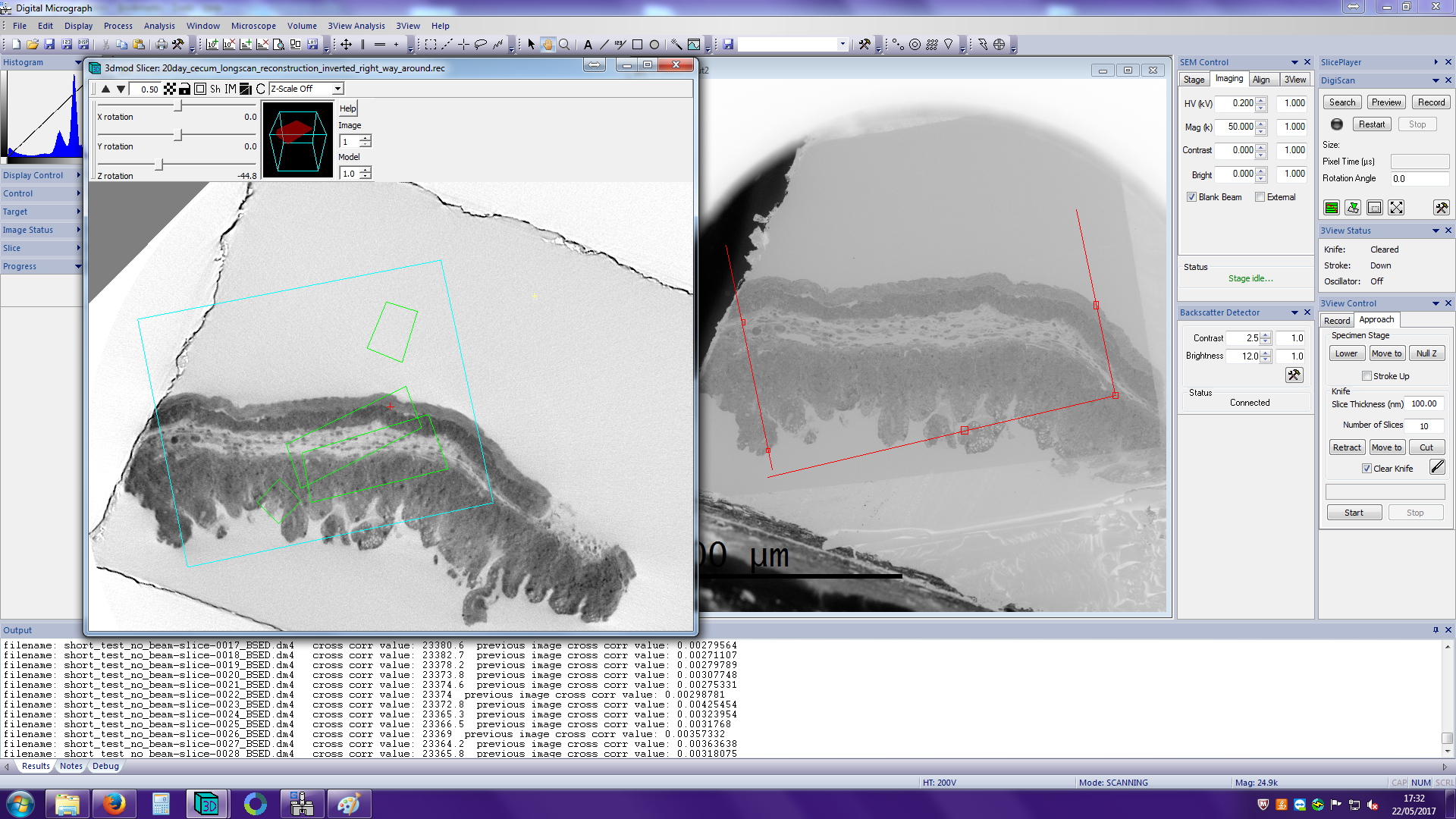


**Figure S1: Using X-ray data to trim resin blocks during sample preparation:** The inverted X-ray data (left window) is opened in IMOD ([http://bio3d.colorado.edu](http://bio3d.colorado.edu/)) to compare with live imaging on the SEM. A box (blue) is defined to enclose the underlying regions of interest (green boxes). On the SEM side the cut-out box can be used to define regions to mark for subsequent trimming (smaller squares in this example).

Here we demonstrate opening the inverted contrast X-ray data in the ‘Slicer’ window of 3dmod, while simultaneously imaging the block-face in the SEM (Fig S1). Using the ‘Slicer’ window allows us to rotate the X-ray data so that the orientation matches the orientation of the EM image. We have used 3dmod to model regions of interest at depth (model mode, middle click to place points: See 3dmod guide for more information on how to make a model (<http://bio3d.colorado.edu/imod/doc/3dmodguide.html>). Once the precise section number of the X-ray data has been identified it is possible to copy the ROI boxes onto the current section (Edit> contour>Copy, then select ‘Copy to section #’ from the pull-down menu). Once the ROIs are plotted on the current section it is possible to draw a box that encloses the hidden ROIs to be used to guide subsequent trimming. Here we have drawn the box in blue in 3dmod (Fig S1, left panel), while copying the same box onto the block-face image using the line drawing tool in Digital micrograph (Fig S1, red lines, right panel).

The traced trim lines can now be used to select specific spots to burn on the block surface. We used a live view window along with the image of the traced lines to accurately move the stage to specific regions on the block-face that match the lines for trimming. Each of these stage positions was saved so that we could return later without the need to view an image to confirm the location.

*Burning the fiducial marks without an ion beam instrument.*

In order to burn the sample surface we set the microscope to 30kV (in order to increase the beam energy), then we set the magnification to 50,000x and took a 2000x2000 image. We did not change the imaging settings as we did not need to see the image produced. We simply used the intense beam to burn a hole into the resin surface.


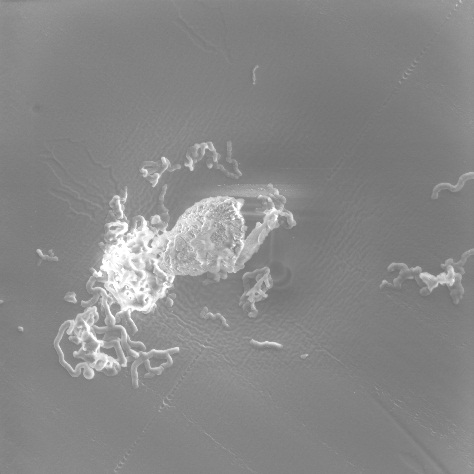

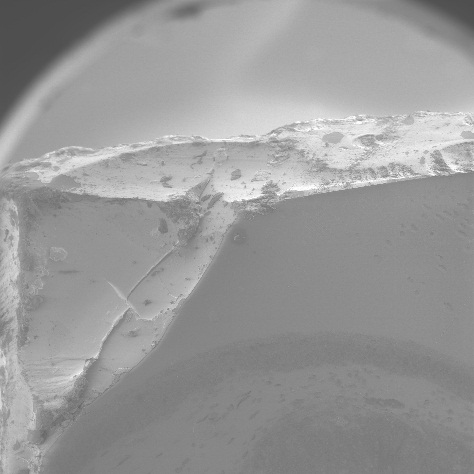


**A**

**B**


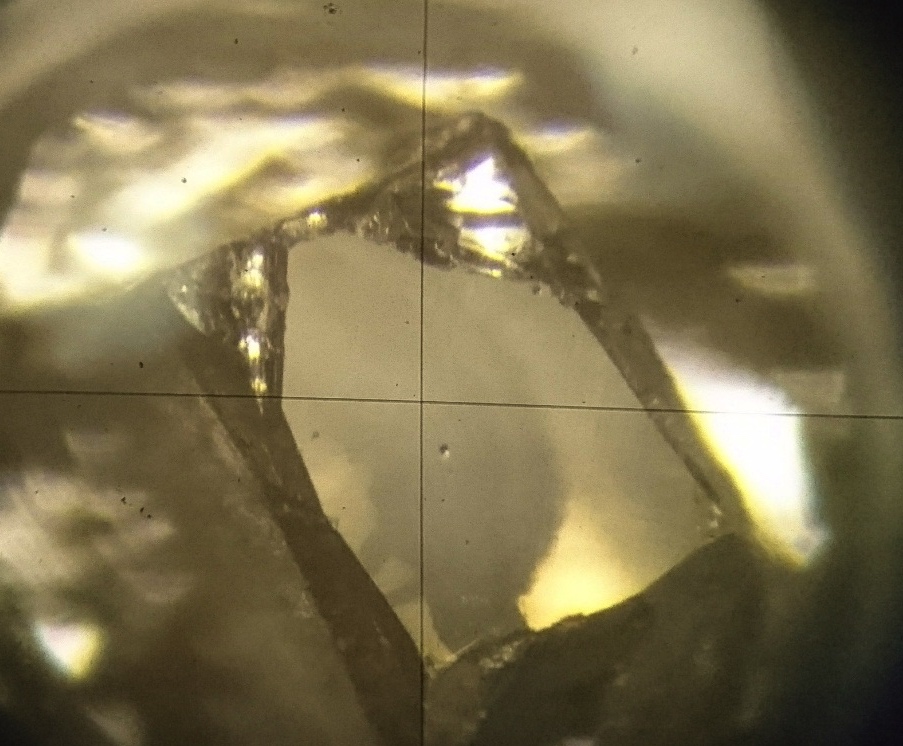


**C**

**Figure S2: Images of marks burnt in the resin surface**. **A**) Secondary electron image of the resin sample deliberately burnt by the beam (red circle). **B**) Higher magnification SEM image of the ‘burnt’ spot showing deformation of the resin surface. **C**) The beam damage can be seen in a dissecting microscope as small marks on the surface (red circle).

By systematically moving the stage to each of the marked positions and marking at 30kV at 50,000x it is possible to mark multiple positions on the resin surface (note that there is no need to focus, or adjust the settings at each position as we are just using the beam to damage the block surface).

If the line to be marked is located in a featureless area of empty resin then it is necessary to touch the surface in order to add debris that can be used to accurately position the markers (Fig S3). The general principle is to take an image of the clean block-face and mark on the lines to trim based on the x-ray data (left panel, Fig S3). Take a note of the magnification and do not move the stage. Now vent the chamber and gently touch the surface of the block with an ungloved finger in order to deposit some finger grease. When the sample is returned to vacuum an identical magnification image can be taken and the lines can be copied and pasted onto the, now contaminated, image. At high magnification there will now be enough contaminants that an accurate stage position can be found, meaning that the line can be marked accurately.


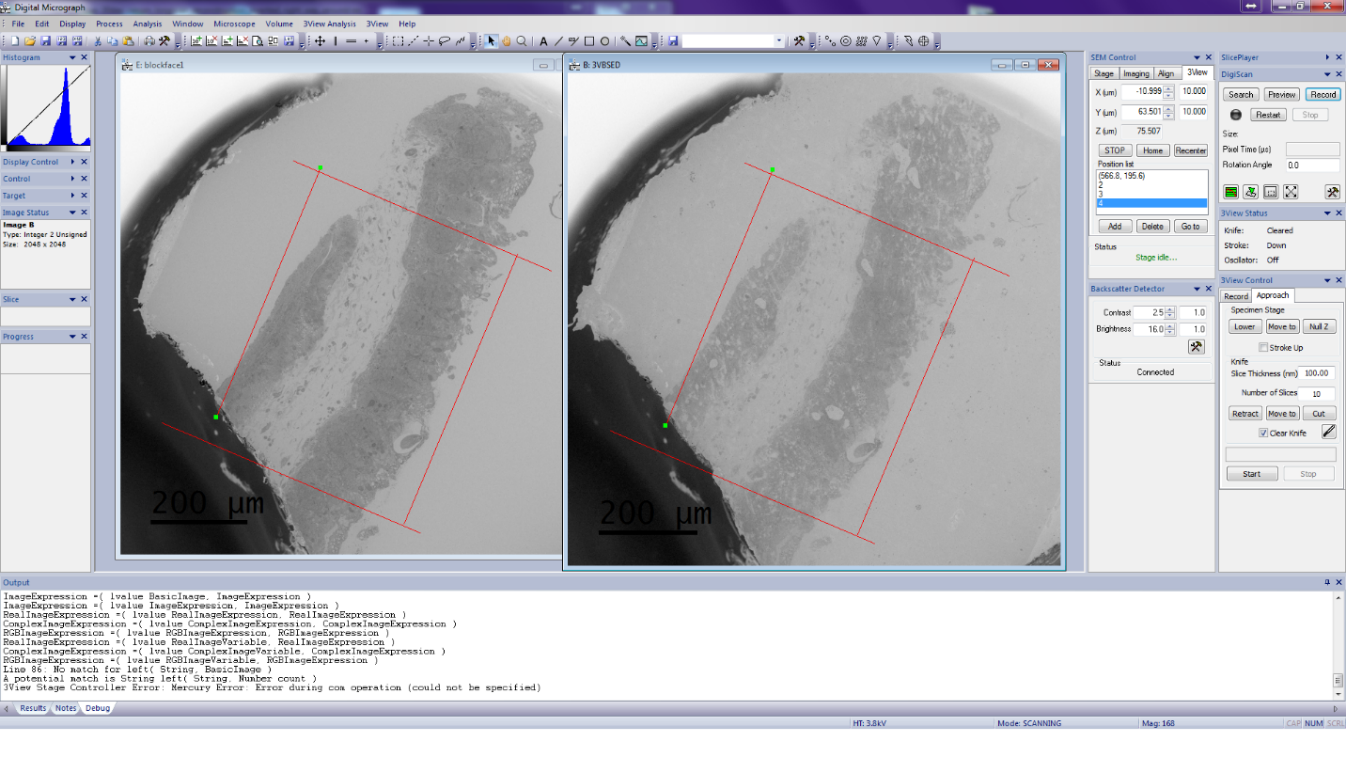


**Figure S3: deliberate contamination of the sample surface can be used to improve accuracy of surface marking**. Image in the left panel is the freshly trimmed block-face imaged with back-scattered electrons showing sample staining. The image in the right panel is the same sample after it has been contaminated with finger grease. The red rectangles indicate the desired size of the block-face; note that the top left corner of the rectangle is within empty resin, making it difficult to mark its position using the beam. The contamination (right panel) can be seen as some bright and dark spots that appear at random on the surface. The pattern of debris on top of the empty resin now allows specific regions to be identified meaning that the block-face can be accurately marked.

*Trimming the sample using markers.*

Once all of the markers have been placed it is possible to visualize these under a dissecting microscope in order to accurately trim the sample (Fig S4). Rather than using a ring illumination we have found that it is more beneficial to use a horizontal illumination that is then reflected onto the block surface. In order to see the marks we reflect the light off the back of the razor blade that we will use to trim the sample. By carefully focusing on the block-face we can slowly lower the out of focus razor blade while reflecting the light to allow us to see the marks. As we approach the sample surface the blade will come into focus, which allows us to accurately position the blade for trimming.

In order to reduce stress on the sample it is best to only shave a small amount off the sample with each cut. By slowly advancing towards the fiducial marks you can remove the unwanted material and you can also test the accuracy of you cutting and adjust this accordingly.


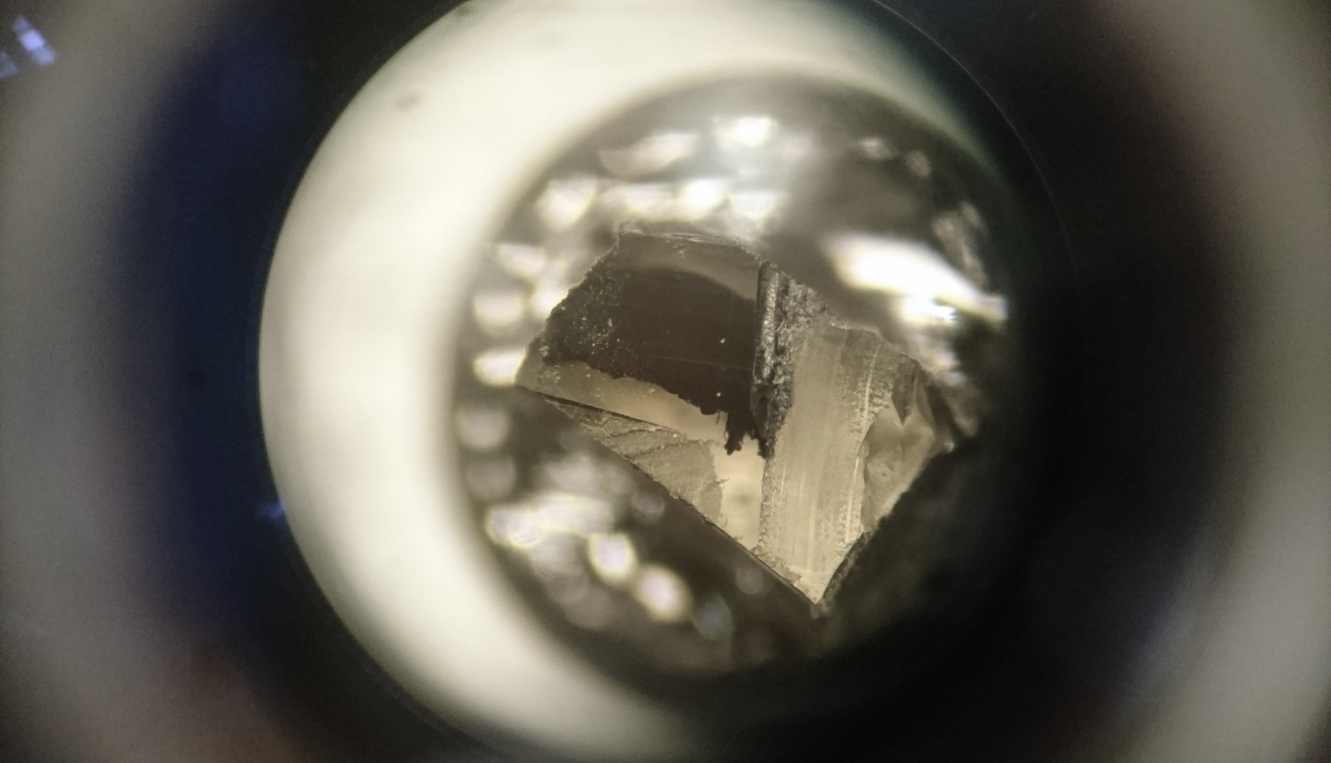

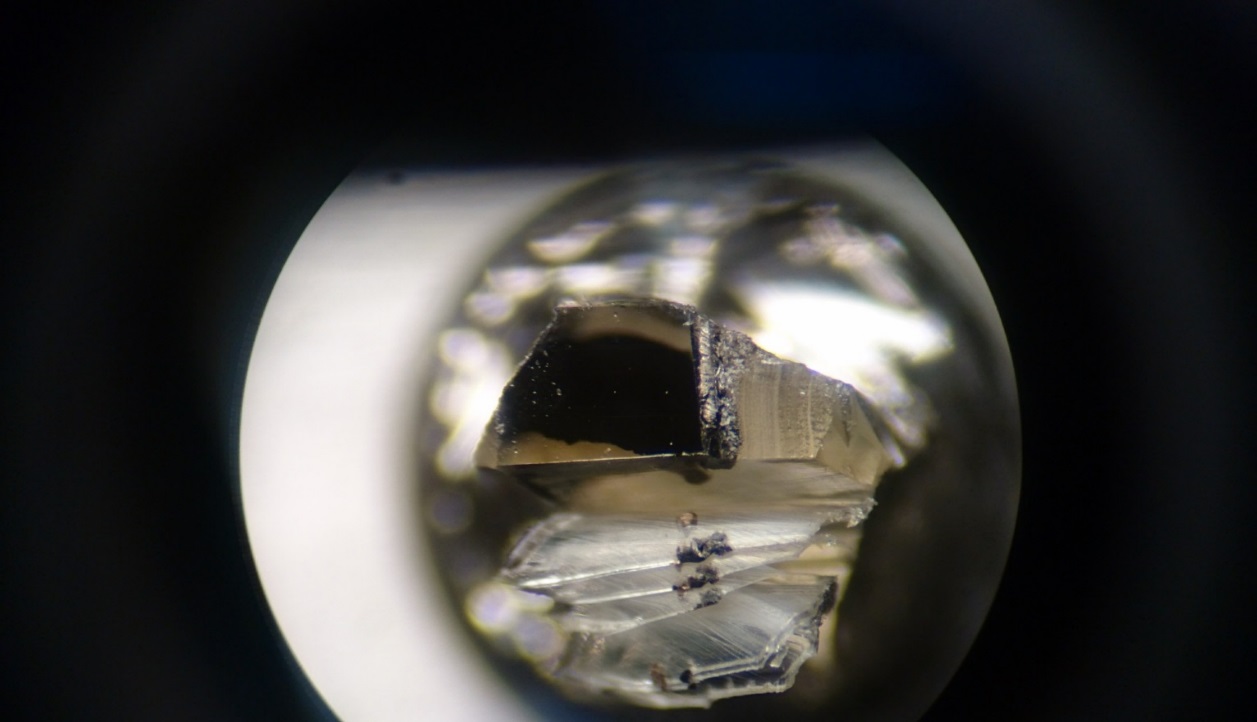


**Figure S4: Trimming the marked sample using a razor blade**. The sample can be trimmed carefully from the point furthest away from the fiducial marks. Thin slices are taken moving closer to the marks on the surface.

# References

[1] J.R. Kremer, D.N. Mastronarde, J.R. McIntosh, Computer visualization of three-dimensional image data using IMOD, J. Struct. Biol. 116 (1996) 71–76. doi:10.1006/jsbi.1996.0013.

[2] D.N. Mastronarde, SerialEM: A Program for Automated Tilt Series Acquisition on Tecnai Microscopes Using Prediction of Specimen Position, Microsc. Microanal. 9 (2003) 1182–1183. doi:10.1017/s1431927603445911.
